# Technological Ecological Momentary Assessment Tools to Study Type 1 Diabetes in Youth: Viewpoint of Methodologies

**DOI:** 10.2196/27027

**Published:** 2021-06-03

**Authors:** Mary Katherine Ray, Alana McMichael, Maria Rivera-Santana, Jacob Noel, Tamara Hershey

**Affiliations:** 1 Department of Psychiatry Washington University in St. Louis St. Louis, MO United States; 2 Department of Psychiatry Mallinckrodt Institute of Radiology Washington University in St. Louis St. Louis, MO United States

**Keywords:** ecological momentary assessment, continuous glucose monitoring, actigraphy, accelerometer, ambulatory blood pressure monitoring, personal digital assistant, mobile phone, smartphone, mHealth

## Abstract

Type 1 diabetes (T1D) is one of the most common chronic childhood diseases, and its prevalence is rapidly increasing. The management of glucose in T1D is challenging, as youth must consider a myriad of factors when making diabetes care decisions. This task often leads to significant hyperglycemia, hypoglycemia, and glucose variability throughout the day, which have been associated with short- and long-term medical complications. At present, most of what is known about each of these complications and the health behaviors that may lead to them have been uncovered in the clinical setting or in laboratory-based research. However, the tools often used in these settings are limited in their ability to capture the dynamic behaviors, feelings, and physiological changes associated with T1D that fluctuate from moment to moment throughout the day. A better understanding of T1D in daily life could potentially aid in the development of interventions to improve diabetes care and mitigate the negative medical consequences associated with it. Therefore, there is a need to measure repeated, real-time, and real-world features of this disease in youth. This approach is known as ecological momentary assessment (EMA), and it has considerable advantages to in-lab research. Thus, this viewpoint aims to describe EMA tools that have been used to collect data in the daily lives of youth with T1D and discuss studies that explored the nuances of T1D in daily life using these methods. This viewpoint focuses on the following EMA methods: continuous glucose monitoring, actigraphy, ambulatory blood pressure monitoring, personal digital assistants, smartphones, and phone-based systems. The viewpoint also discusses the benefits of using EMA methods to collect important data that might not otherwise be collected in the laboratory and the limitations of each tool, future directions of the field, and possible clinical implications for their use.

## General Introduction

Type 1 diabetes (T1D) is one of the most common chronic childhood diseases with a rapidly rising incidence and prevalence [1,2]. It is an autoimmune disease characterized by the loss of insulin-producing β cells of the pancreas, leading to an inability to use glucose as fuel, thus requiring life-saving exogenous insulin administration [3]. The management of glucose is challenging because patients must consider a myriad of factors when making diabetes care decisions (eg, insulin administration and glucose checks), such as the amount of carbohydrates consumed, insulin administered, physical activity, stress, illness, hormonal changes that cause natural spikes in glucose (eg, *dawn phenomenon*), and access to diabetes treatment technologies (eg, continuous glucose monitoring [CGM] and insulin pump) [4-6]. This complex web of factors and subsequent management decisions can lead to a significant amount of glucose variability throughout the day, with frequent fluctuations between normal (euglycemia), high (hyperglycemia), and low (hypoglycemia) glucose levels [4]. In fact, research has shown that young children with T1D are in a hyperglycemic state approximately 50% of the time [7] and experience thousands of hypoglycemic events in their lifetime [8]. These types of glycemic states have been shown to be associated with short- and long-term complications, including, but not limited to, hypoglycemia (sometimes severe), diabetic ketoacidosis (DKA), heart disease, retinopathy, nephropathy, neuropathy, sleep disturbances, and cognitive impairments [9-11].

At present, most of what is known about each of these complications and the health behaviors that may lead to them have been uncovered in the clinical setting or laboratory-based research using medical record data extraction, retrospective interviews asking participants to recall information from past events, and tests that measure health over extended periods (eg, hemoglobin A_1c_ [HbA_1c_]). However, there are known limitations with measurements of this type, given their inability to capture the dynamic behaviors, feelings, and physiological changes associated with T1D that fluctuate from moment to moment throughout the day. Importantly, these measurements can also be confounded by recall bias [12], thus limiting our capacity to characterize and understand T1D. A better understanding of T1D in daily life could potentially aid in the development of behavioral and pharmaceutical interventions to improve diabetes care and mitigate the negative medical complications often associated with T1D. Therefore, there is a need to measure repeated, real-time, real-world features of T1D in daily life. This approach, known as ecological momentary assessment (EMA), can include a variety of methods, such as handheld computers, diaries, phones, smartphones, activity trackers, and physiological monitors (eg, CGM and ambulatory blood pressure monitoring [ABPM]) [12]. These methods can provide more valid ecological representations of patients’ experiences, behaviors, and physiological measures with in-the-moment collection of information [12]. Fortunately, the widespread use of EMA is becoming more feasible because of the lower cost, greater familiarity, and usefulness of ambulatory technological devices such as smartphones and activity trackers (eg, Fitbit), especially in youth [13-15]. As a result, the use of wearable devices to collect real-world data is becoming increasingly popular across a variety of scientific fields to better understand disease states in a person’s natural environment [16].

The use of EMA to understand factors related to T1D in daily life may be particularly important in youth, as they typically have more challenges with glycemic control than other age groups with diabetes [17] and are reaching developmental milestones that could impact their daily glucose management behaviors. For example, youth may behave differently with their T1D care if they are in a group of peers they feel the need to fit in with, or act differently, as they begin to seek independence from their parents [18]. Furthermore, it has been suggested that youth may not have yet developed the full cognitive abilities needed to integrate the information and skills needed to make appropriate management decisions [19]. This limitation could be even more concerning in patients with T1D, as a rich body of the literature has shown that acute complications (eg, severe hypoglycemia and DKA) and chronic hyperglycemia measured in the laboratory are associated with lower cognitive scores [11]. Furthermore, youth may still be unburdened by medical complications that often accompany T1D later in life. EMAs could potentially provide a more sensitive tool for early tracking of T1D complications that may not yet be detected in a clinical setting. Taken together, EMA methods could provide insight into important daily factors that influence T1D care behaviors and track physiological changes that may be predictive of complications in this particularly vulnerable population.

This viewpoint aims to describe EMA tools that have been used to collect data in the daily lives of youth with T1D and discuss studies that explored the nuances of T1D in daily life. This viewpoint focuses on several methods used to assess behavioral and physiological measures, including CGM, actigraphy, ABPM, personal digital assistants (PDAs), smartphones, and phone-based systems. The viewpoint also includes a discussion of the benefits of using EMA methods to collect important data that might not otherwise be collected in the laboratory and the limitations of each tool, future directions of the field, and possible clinical implications of using these tools.

## Overview of Methods Used to Identify and Assess EMA Tools

The following keywords were searched using the PubMed database between January and September 2020: (“type 1 diabetes” and “ecological momentary assessment”), (“type 1 diabetes” and “EMA“), (“type 1 diabetes” and “actigraphy”), (“type 1 diabetes” and “field study”), (“type 1 diabetes” and “smartphone app”), (“type 1 diabetes” and “smartphone”), (“type 1 diabetes” and “phone”), (“type 1 diabetes” and “personal digital assistant”), (“type 1 diabetes” and “PDA”), (“type 1 diabetes” and “mHealth”), (“type 1 diabetes” and “mobile health”), (“type 1 diabetes” and “wearable”), (“type 1 diabetes” and “electronic health”), (“type 1 diabetes” and “eHealth”), (“type 1 diabetes” and “sleep”), (“type 1 diabetes” and “ambulatory”), (“type 1 diabetes” and “accelerometer”), (“type 1 diabetes” and “CGM”). Each pair of search terms was combined with “youth,” “adolescent,” and “children.” Articles were reviewed starting in 2005, given the rapid development of technology, particularly real-time CGM, around this year.

To narrow the focus of the viewpoint, articles for the *literature review* portion of each section were not typically included in our synthesis of the literature if youth with T1D were not the primary study population (eg, articles combining all youth and adult data and articles focused on the caregivers of youth with T1D); the article was a review, case report, book chapter, editorial, conference abstract, study protocol, or comment; the article was written in a language other than English; the study was conducted in an animal model; the article did not directly seek to obtain EMA measures multiple times per day on a technological device; the study was conducted entirely in an in-lab setting; physical activity was the primary focus of an actigraphy study; or the article focused on flash glucose monitoring versus CGM. This viewpoint was intended to provide an overview of popular EMA tools to collect data in youth with T1D and provide a generally comprehensive but not exhaustive discussion of the articles that have used each tool.

## Viewpoint of Methodologies

### Continuous Glucose Monitoring

#### Introduction

It is becoming increasingly clear that HbA_1c_ has many limitations and is not the only important factor for measuring glycemic control and predicting the risk of medical complications in T1D [20]. Although glucose variability remains poorly understood, it has recently been highlighted as a potential risk factor for developing disease complications [21]. Understanding the importance of glucose variability is becoming much more feasible with the use of CGM technology, perhaps the most well-known EMA method used in T1D. Real-time CGMs typically measure interstitial glucose every 1-5 minutes via a sensor embedded under the skin, and information is then transmitted to a tracking device such as a smartphone app, receiver, or insulin pump [22-24]. Most current CGMs usually measure glucose every 5 minutes, with 288 measurements per day [24] compared with self-monitored blood glucose (SMBG), which is often only measured to test for hypoglycemia or in situations such as with meals, exercise, bedtime, or certain tasks (eg, driving). This can vary among participants but typically requires SMBG measurements 6-10 times per day [25]. Thus, the frequent testing of glucose with CGM provides a more comprehensive view of glucose patterns and variability over time compared with glucose snapshots provided by SMBG or HbA_1c_. Data collected via CGM can provide information on glucose trends, the amount of time a person is in a specified glucose range (eg, euglycemia: 70-180 mg/dL), asymptomatic glycemic events, the amount of glycemic variability, the mean glucose over discrete periods (eg, 14 days), overnight glucose patterns—not typically captured with SMBG—and postprandial glucose peaks [22,25-29].

This more encompassing view of glucose patterns can aid health care providers in the development of optimized goals and plans aimed at improving glucose levels and mitigating negative medical complications [20]. CGM has also been shown to be *empowering* for youth with T1D, as it allows them to easily access data such that they can have more control over their glucose, provides motivation for dietary intake changes, and helps them manage hyper- and hypoglycemia [30]. As a result of these benefits, the use of CGM is increasing rapidly, especially in young children [31-33]. For example, CGM use in youth in a large diabetes registry increased from 4% to 31% between 2013 and 2017 [33].

#### Literature Review

##### Using CGM to Identify Relationships Between Daily Factors and T1D Symptoms and Behaviors

CGM data have been used to better help understand the relationship between daily activities, behaviors, and glucose control including diets and eating patterns [34-39], exercise or activities [40-50], sleep [51-53], amount of time spent at home (eg, before and during the COVID-19 lockdown) [54], and externalizing behaviors [55].

##### Using CGM to Measure Outcomes

Data collected with CGM have also provided an important tool for testing the feasibility and effectiveness of insulin delivery systems, insulin treatments, adjunctive diabetes medications, T1D screening, new glucose monitoring systems, algorithms for the promotion of improved glycemic control, sensor-augmented therapy algorithms, and closed-loop systems [56-91]; diabetes alert dogs [92]; education programs aimed at improving impaired hypoglycemia unawareness, daily therapy decisions, and cardiovascular health [93-95]; and use of glucose sharing data with others [96]. This technology has also been used to assess the relationship between continuous glucose measures and other diabetes-related outcomes, including long-term glycemic control, dysglycemia [97,98], future T1D diagnosis or dysglycemia in preclinical youth [99-101], HbA_1c_ [102-105], C-peptide [102], insulin sensitivity [106], severe hypoglycemia [107], time in target range [108-110], glucose variability [109-113], detection of hypo- or hyperglycemia [108-110,114-116], glycated albumin [104], fructosamine [104], and 1,5-anhydroglucitrol [104]. Furthermore, CGM has been used to determine the relationship between continuous glucose measures and other medical outcomes, including body composition [117], markers of inflammation [118], cardiovascular health [119], and brain health (eg, white matter integrity) [120].

##### Using CGM to Improve T1D

Thus far, there is substantial evidence to suggest that CGM use is associated with improved glycemic control, including lower or improved HbA_1c_, reaching target HbA_1c_ [121-146], decreased hyper- or hypoglycemic events or time in a hyper- or hypoglycemic state [137,138,145-148], reduced SD or mean glucose [134,146], reduced glucose variability [134,137,148], and increased time in the target glucose range [127,137,146]. CGM use has also been shown to be related to improved treatment and outcomes, including more advanced and optimized treatment recommendations by physicians to improve glycemic outcomes [149,150], increased satisfaction with diabetes treatment [123,151], improved perceived awareness of or hormonal responses to hypoglycemia [123,152], reduced fear of hypoglycemia [153,154], reduced patient distress [139], and altered amount of insulin used per day (eg, both decreased and increased insulin use per day) [140,154]. Outside of direct diabetes outcomes, CGM use has been shown to be associated with a higher quality of life (when a new algorithm was used to help guide insulin administration) [155], improved school attendance [151], and increased comfort with diabetes management in the school setting [156].

Furthermore, studies have shown significant benefits of using CGM sensor-augmented pump therapy systems that can initiate automatic functions for suspending insulin in the event of current or predicted low-glucose and closed-loop systems. For example, predictive low-glucose suspension or hyperglycemia and hypoglycemia minimization functions have been shown to be related to improvements in HbA_1c_ [89], greater time in range [74], reduced mean glucose levels in the morning [74], reduced area under the curve for hypoglycemia and hyperglycemia (eg, >240 mg/dL), and hypoglycemia [67,69,70]. Closed-loop systems have also been shown to improve T1D, including lower mean glucose [60,75,76,80,86], reduced frequency of hypoglycemia intervention [60,80], lower proportion of time or less time in hypoglycemia [68,72,80], and increased time in range or time in tight range of control (80-140 mg/dL) [68,75,76,85,86]. Furthermore, youth have expressed positive experiences with closed-loop systems, including self-reported positive impact on sleep, their routines, and safety [157]. Interestingly, a study evaluated the ability of a heart rate monitor to inform closed-loop system decisions with exercise and found that the incorporation of heart rate data into the closed-loop system improved time below a glucose level of 70 mg/dL even though it did not reduce the number of hypoglycemic events [48]. When comparing the 2 types of systems, studies have shown significant benefits for closed-loop systems versus sensor-augmented therapies with glucose suspend functions. For example, youth with closed-loop systems or control-to-range algorithms have been shown to have less time below the target range [71], reduced symptomatic hypoglycemia at night [71], increased time in range [75], and lower mean glucose [75].

Although there is a significant amount of evidence showing the benefits of CGM in improving T1D, it is important to note that not all studies have found statistically significant improvements in all measures between CGM users and CGM nonusers or with the initiation of CGM use across different time points and age groups [126,129,138,140,148,153,154,158-166]. The lack of evidence for the benefits of CGM could potentially be the result of the amount of time spent using the tool. For example, more and consistent use versus intermittent use, full compliance with the research protocol, age, baseline glycemic control, and self-efficacy with CGM use have all been suggested to be important factors for clinical improvements with CGM [56,122,124,131,132,134,140,148,159,161,167-169].

#### Limitations of CGM

It is clear that CGM has many advantages; however, there are limitations to its use. There is still the potential for measurement error, false alarms (eg, alert for hypoglycemia when the patient is euglycemic), alarms that do not awake patients at night [24,170-173], signaling loss-driven gaps in data, and data interpretation challenges [174]. The sensors can also be uncomfortable or painful or fall out, which is of significant concern given the high cost of the technology and difficulties with insurance coverage [24,171,173-179]. There can also be an annoyance or *alarm fatigue* with frequent alerts about glucose and sensor status, potentially causing patients to feel overwhelmed or embarrassed, especially in public environments [28,171,172,177,180-182]. Furthermore, the abundance of information provided by CGMs may cause anxiety, frustration, and self-thoughts of failure or that they have *bad* blood glucose patterns, especially if they have difficulty interpreting the substantial amount of data provided by CGM [24,28,174]. In the same vein, youth may fear chastisement from others (eg, parents and physicians) if they have frequent hypo- or hyperglycemic events that would not otherwise be observed with SMBG [24]. All these factors could significantly affect the adherence to CGM use. However, programs are being developed to help better teach youth how to overcome the challenges associated with CGM [183].

#### CGM Conclusions

Overall, frequent, repeated glucose measurements with CGM can provide great benefits over one-time snapshots of glucose. In addition to a significant enrichment of glucose information, CGMs could further enhance our knowledge of T1D when paired with other EMA methods to determine how in-the-moment behaviors are associated with subsequent glucose patterns and how glucose patterns relate to daily health (eg, sleep). CGM is growing in popularity among youth with T1D and has significant advantages over in-lab glucose measures or SMBG, as it provides a wealth of knowledge that can be used to better understand glucose fluctuations. However, there are still limitations to its use, such as inaccurate readings and discomfort, that need to be addressed in future technological development.

### Actigraphy

#### Introduction

Actigraphy is a noninvasive, motion-sensing tool often worn on the wrist for extended periods to collect information about measures such as physical activity or sleep [184]. Sleep is of particular concern for youth with T1D, as research has shown consistent sleep disturbances compared with their peers without T1D [10], with an average of approximately 26 minutes less sleep per night [185]. Actigraphy provides numerous advantages over in-lab methods of collecting sleep data, as it is inexpensive and can be used to examine sleep patterns over multiple days [186-188]. Furthermore, it is relatively suitable for correctly identifying sleep periods in youth and can assess numerous facets of sleep (eg, sleep onset, sleep onset latency, frequency of nocturnal waking, duration of nocturnal waking, wake after onset, the midpoint of sleep, nap duration, total sleep period, wake time, total sleep time, sleep efficiency, fragmentation of sleep, and longest continuous sleep episodes) by repeatedly measuring movement and heart rate (with some, but not all trackers) [188]. Taken together, actigraphy offers an affordable and convenient method of measuring sleep at home, providing a unique means of collecting more information about how sleep is affected in the daily lives of youth with T1D.

#### Literature Review

##### Using Actigraphy to Identify Relationships Between Daily Factors and T1D Symptoms and Behaviors

Actigraphy has uncovered important relationships between sleep and diabetes care behaviors in daily life. For example, both average sleep duration and sleep variability have been shown to be associated with diabetes care behaviors (eg, less average sleep duration related to decreased SMBG or more sleep variability related to decreased SMBG) [189,190]. Furthermore, actigraphy-derived data have shown a relationship between HbA_1c_ and altered sleep (eg, more total sleep time related to lower HbA_1c_) [51,189], found a relationship between sleep in children and sleep in their parents [191], and showed that youth with T1D who have obesity may have different sleep patterns compared with patients with T1D without obesity [192]. Importantly, actigraphy has uncovered relationships between specific patterns of glucose control (eg, greater glucose variability, more time in hypoglycemia, and suboptimal HbA_1c_) and disrupted or variable sleep [53,193-195]. For example, Monzon et al [193] found that measures of sleep (eg, sleep onset latency and nighttime awakenings) predicted more variability in glucose on weekend days, highlighting the potential importance of maintaining consistent sleep routines throughout the week.

It is important to note that most of these studies were correlational and therefore could not determine the directionality of the relationship between sleep and glycemic control [51,53,189,190,195]. It has been suggested that there is bidirectionality in the relationship (eg, more glycemia outside of the recommended range increases the likelihood of hypoglycemia and hyperglycemia at night, which can then impair sleep [190]). Monzon et al [193] found a bidirectional association between glucose variability and awakenings on weekend nights, suggesting that youth with more weekend awakenings may have more glycemic variability in the following days and that youth may have more awakenings after weekend days where they have more glucose variability. They also suggest that future research considers how both physiology (eg, physiological changes in glucose) and behavior (eg, waking to check glucose at night) could explain the bidirectional relationship between glucose control and sleep [193].

##### Using Actigraphy to Measure Outcomes

Given that youth with T1D often have sleep impairments or do not meet current sleep recommendations [51,189,190,193-196], interventional strategies are needed to help improve sleep in this population; 2 recent studies found that smartphone app–based interventions improved sleep measured via actigraphy in youth with T1D. Jaser et al [197] found that their intervention improved sleep efficiency and increased total sleep time by approximately 48 minutes. Similarly, Perfect et al [198] found that their sleep intervention protocol increased actigraphy-measured sleep by approximately 29 minutes. These studies highlight the promise of mobile health (mHealth) interventions (ie, medical care supported by a mobile device) [199] to treat sleep in this vulnerable population. Furthermore, not only is actigraphy being used to assess whether these mHealth interventions can improve sleep, but it is also being used to determine whether new diabetes management systems (eg, hybrid closed-loop systems) can improve sleep among youth with T1D and their parents [200].

#### Limitations of Actigraphy

It is important to note that although actigraphy has been shown to be relatively sensitive in measuring sleep, not all studies have shown comparative results between actigraphy and the gold standard of sleep measurement—polysomnography—and it has been shown that actigraphy has low specificity for certain measures such as detection of wake after sleep onset [188]. It has been suggested that actigraphy may be a better tool for collecting typical sleep data and may be less reliable in adequately measuring disturbed sleep [186]. Furthermore, studies have used a wide range of tracking devices (eg, Fitbits and Actigraphs), which could potentially create discrepancies between results [188]. More tool development and validation testing are needed to measure sleep adequately, especially disrupted sleep.

#### Actigraphy Conclusions

Actigraphy is increasingly being used to assess sleep in youth with T1D. The EMA tool provides an inexpensive way to objectively measure sleep in the daily lives of youth in their natural environments. Studies have shown more sleep disturbances in youth with T1D, and these disturbances have also been shown to be related to important T1D self-care behaviors and glycemic control. Promising preliminary studies have shown the potential use of app-based interventions to improve sleep; however, more trials are needed. Furthermore, more validation testing and consistent tracker use should be considered in future studies.

### Ambulatory Blood Pressure Monitoring

#### Introduction

Cardiovascular disease is a significant concern in patients with T1D. Unfortunately, in-office blood pressure measurements only capture a snapshot of blood pressure, which changes continuously throughout the day [201]. In-office monitoring is also influenced by masked hypertension (patients can have normal in-office blood pressure but elevated when measured 24 hours per day out of the office) and the white-coat phenomenon (patients have elevated blood pressure when measured in the office but have normal blood pressure in daily life) [202]. Specific to youth with T1D, 9.5% have been shown to have masked high blood pressure, whereas 32% have been shown to have white-coat hypertension [203]. To mitigate these phenomena and better assess the impact of circadian rhythms on blood pressure, researchers and clinicians have turned to ABPM as a tool to collect EMA data on heart function [202,204,205]. Importantly, a more accurate determination of heart function using the EMA tool could lead to a more appropriate use of blood pressure medications [206]. ABPM is typically measured via an ambulatory blood pressure cuff every 15-20 minutes during the day and every 20-30 minutes at night (varying by study), providing a large amount of information on blood pressure and heart rate variability in varying contexts [201].

#### Literature Review

##### Using ABPM to Measure Outcomes

A large number of studies have used ABPM to measure cardiovascular function outcomes in youth with T1D and found that patients often have high blood pressure measures (eg, high systolic or diastolic pressure at different times of day), prehypertension, or higher blood pressure than their peers who do not have T1D [203,207-225], with higher blood pressure–measured with ABPM being predictive of future hypertension [220]. Many factors have been found to be associated with measures of high blood pressure in this population, including higher HbA_1c_ [208,209,215,226], age [209], sex [208,209], diabetes duration [208,209,214,225,226], unstable glycemic control [225], insulin dose [209,215,225], BMI [209,215,226], genetics [227,228], triglycerides [214], high salt intake [226], and selectins involved in inflammation (eg, E-selectin) [214,229].

People without T1D typically have a dip in nocturnal systolic and diastolic blood pressure by approximately 10%-20% from daytime blood pressure [201]. However, research using ABPM has consistently shown that patients with T1D often do not have this normal *dip* or have less dipping than youth without T1D [207,209,218,221,222,224,230-234]. This abnormal dipping has been shown to be related to age, sex (ie, being female associated with more abnormal dipping) [209], HbA_1c_ [209,226], higher ambulatory arterial stiffness—a potential marker of arterial stiffness to predict heart mortality [232], reduced 24-hour heart rate [233,234], reduced mean daytime heart rate [233,234], prolonged QT interval (interval from the Q to the T electrical wave), greater left ventricular end-diastolic and end-systolic diameters [233,234], greater left ventricular mass index [234], and specific heart oscillation patterns [235]. In addition, not only does high blood pressure present a problem in itself, but it is also associated with other negative medical complications. In fact, several studies have found a relationship between high blood pressure measures obtained with ABPM and markers of kidney damage or disease in youth with T1D [211,215,220,222,236-239].

Although there is substantial evidence for increased blood pressure in youth with T1D, not all studies have found robust, statistically significant relationships between diabetes and high blood pressure across contexts [240,241]. For example, Raes et al [240] found no significant difference in blood pressure between youth with T1D and those without T1D when participants were at rest but observed significantly higher blood pressure in patients with T1D while they were participating in exercise. Furthermore, not all studies have found a relationship between blood pressure and markers of kidney damage [218,242]. For example, Soltysiak et al [242] found no relationship between blood pressure and increases in neutrophil gelatinase-associated lipocalin, an early marker of kidney damage.

#### Limitations of ABPM

ABPM use in research is limited by the potential interference it presents with a patient’s activities in daily life and inaccurate readings if the cuff is placed incorrectly during measurement [243]. Therefore, more research is needed to develop less intrusive and more valid devices or protocols. Furthermore, the nocturnal dipping status may not be consistently reproducible. Thus, it has been suggested that the focus of research is on 24-hour systolic blood pressure versus dipping status [206].

#### ABPM Conclusions

ABPM provides an excellent tool for measuring real-world heart function while avoiding potentially confounding variables, such as the white-coat phenomenon and masked hypertension, which might otherwise conceal heart function complications when measured in a laboratory setting. The use of this EMA method has uncovered important heart function complications in youth with T1D. Overall, blood pressure measurements obtained from ABPM have consistently been shown to be elevated in youth with T1D, and this increased blood pressure has been associated with markers of kidney damage. Thus, real-world testing could highlight the need to conduct earlier tracking of heart function and interventions in youth with T1D.

### Personal Digital Assistant

#### Introduction

All of the EMA tools discussed so far have passively collected repeated, real-time data such that the patients did not consciously and purposefully have to interact with the device. To collect more information about a patient’s conscious behavior or perceived experiences, more active EMA methods are needed, such as PDAs. PDAs are often referred to as handheld computers, as they can connect to the internet, be used to organize information, and communicate via email or a personal computer [244]. Although PDAs can function similarly to more recent technological developments (eg, smartphones), supplying PDAs to participants could help reduce limitations related to patients not having their own devices or owning devices that are not compatible with the study software.

#### Literature Review

##### Using PDAs to Identify Relationships Between Daily Factors and T1D Symptoms and Behaviors

Several studies have been conducted using PDAs to determine how environmental factors impact patient experiences and diabetes care behaviors in youth with T1D in real-world settings. For example, Helgeson et al [18] had patients complete periodic measures of interpersonal interactions (eg, had the participant had a social interaction? Was the interaction positive or negative?) and mood on a PDA throughout the day and found that less enjoyment and being upset from a social interaction predicted depressed mood and anxiety. PDA devices also found that patients were more likely to check their glucose when they reported a strong desire to blend in with their companions and less likely to check glucose when they wanted to impress people [245]. Taken together, the results suggest that peer relationships in daily life are associated with self-care behavior and psychological well-being in youth with T1D.

As described in the *General Introduction*, lower cognitive scores have been shown to be associated with severe hypoglycemic events, DKA, and chronic hyperglycemia when measured in a laboratory setting [11]; thus, it is important to understand how momentary cognitive function is related to T1D in daily life. Gonder-Fredrick et al [246] had patients use a PDA device to determine the relationship between SMBG and cognitive function (ie, mental math and reaction time task) in a real-world setting. Performance was worse during periods of hypoglycemia (<54 mg/dL) and hyperglycemia (>400 mg/dL) compared with euglycemia (although not statistically different for hyperglycemia and the reaction time task). Thus, mental efficiency is altered by hypo- and hyperglycemia in the daily lives of youths with T1D [246]. Importantly, it is possible that cognitive impairment is not only affected by glucose levels but may also affect subsequent glucose levels through poor T1D care decisions. More research is needed to better understand the complex relationship between glucose levels, cognitive impairment, and T1D care decisions.

##### Using PDAs to Measure Outcomes

PDA devices have also provided important information on T1D outcomes in daily life. Gonder-Fredrick et al [247] assessed real-world glucose symptoms in which youths were asked to estimate their current blood sugar and rate symptoms on a PDA (with the help of a parent) immediately before measuring SMBG. The youths struggled to correctly estimate glucose, making clinically accurate estimates less than one-third of the time and did not detect >40% of hypoglycemic episodes. Children less accurate in detecting low glucose levels also had more severe low blood sugar levels in the subsequent 6-month period. This inability to recognize hypoglycemia is problematic and suggests that more education may be needed to improve the detection of low glucose levels in this population [247].

#### Limitations of PDA

As with most EMA methods that require self-report, there can be uncertainty about the validity of the data, given that they are collected without researcher or clinician supervision [248]. Therefore, it is possible that someone other than the participant is completing the measure or that the patient is not fully dedicated to the task at hand. With the technological nature of PDAs, there is always the risk of software and hardware issues [12] (eg, poor connection to the system transmitting the data). Furthermore, as with most EMA studies, there is a significant concern with testing compliance [12] and dropping out when asked to use these devices in everyday life*.* Finally, PDAs may now be less available as they have been replaced with smartphones.

#### PDA Conclusions

PDAs have captured complex relationships and nuances that could significantly impact a patient’s daily life, which might otherwise be lost if measured only in a clinical or laboratory setting. Several psychosocial and contextual factors have been found to be associated with T1D self-care behavior and psychological well-being, such as wanting to fit in and wanting to impress others. Research has also revealed information about T1D symptoms in the real world, including undetected hypoglycemia and impaired cognitive function. Information collected via PDAs could potentially help provide an avenue for directly addressing these factors to improve T1D management and treatment.

### Smartphone Apps and Phone-Based Systems

#### Introduction

The use of phones to collect EMA data is becoming more feasible as their ownership in childhood and adolescence continues to increase. In those aged 8-12 years, phone ownership increased from 24% to 41% between 2015 and 2019, and in those aged 13-18 years, ownership increased from 67% to 84% [14]. Phone ownership has been shown to be even more common in youth with T1D, with a study reporting that over 92% of patients aged ≥12 years (n=279) carry a phone in their daily lives [249]. The increased feasibility provides an ideal window for the development of new assessment platforms using these tools. As with PDAs, phones can collect data in a variety of ways. For example, they can prompt participants with a notification to enter information at fixed or random intervals throughout the day, or patients could be instructed to upload diabetes information when applicable (eg, when they miss an insulin dose, they tell an app what was happening in their lives at the moment that led to the missed dose).

#### Literature Review

##### Using Smartphone Apps and Phone-Based Systems to Identify Relationships Between Daily Factors and T1D Symptoms and Behaviors

Researchers have developed several smartphone apps to collect EMAs in pursuit of assessing environmental factors related to T1D care behaviors. For example, the *MyDay* smartphone app developed by Mulvaney et al [250] was used to identify psychosocial and contextual factors that impact self-care behaviors (eg, SMBG and insulin administration) in youth with T1D and found that patients reported significant social and contextual barriers to T1D management, including being with family, friends, and alone, fatigue, hunger, having fun, and being in a rush. Importantly, some of the barriers were shown to be significantly associated with self-care behaviors (eg, fatigue was associated with more missed insulin administration) [250], highlighting the potential for tailored treatments to address specific environmental barriers.

Several apps and systems have also been developed that synchronize cellphones to glucometers, allowing patients to upload glucose readings to their phones (eg, SMBG and CGM readings) or synchronize their glucose to their parents’ phones. These apps and systems have also been designed to include features that allow a patient to manually log blood glucose, carbohydrate intake, meals, exercise, medications, insulin pump basal and bolus settings, glucose trends, illness, or life events (eg, vacation), often providing users with an integrated view of the myriad of daily factors that could affect glycemic control. Several of the apps include chat rooms for youth with T1D to communicate with one another, functions that allow patients to contact health care providers, reminders for patients to participate in T1D self-care behaviors, insulin and carbohydrate calculators, tips for diabetes treatment, information about T1D, and gamification incentives for participating in diabetes self-care behaviors [19,251-266].

For example, the *DiaMob* app was partly developed to help patients understand carbohydrate counting and insulin dosing. Participants were asked to provide information about physical activity around mealtimes and the amount of insulin needed to account for carbohydrate intake and capture a photograph of their food. Glucose data could also be integrated to provide an all-inclusive app interface with information about glucose levels, activity, insulin dosage, and meal composition. The results revealed that patients consumed more carbohydrates than they expected and, in the beginning, miscalculated the insulin dosage required to account for carbohydrate intake. Patients found the app supportive for diabetes management, feeling that observing their food made them more mindful of their eating habits and helped them calculate carbohydrates more accurately [19]. A previous study using the app found that participants thought the pictures, physical activity information, integration of pre- and postprandial glucose measurements, and insulin dosages helped them develop a better understanding of how those factors impact glucose measurements [251].

Text message– and phone call–based systems have also been used to collect EMA [252,267-269]. For example, Warnick et al [269] recently tested the accuracy of self-reported SMBG and identified in-the-moment factors that create barriers and motivation for SMBG checks via text surveys. They found that only 39.6% of self-reported SMBG values were accurate, with health being a motivator for SMBG checks, whereas forgetting, not having their devices, and ignoring diabetes tasks were reported as barriers [269]. Furthermore, phone call–based systems have been developed to determine what real-life dietary factors (eg, carbohydrate intake, fiber intake, and physical activity) impact glucose fluctuations and diabetes self-care behaviors (eg, SMBG checks and insulin administration) [270,271]. For example, Mulvaney et al [271] used an automated, interactive touch-tone telephone response system to determine the environmental factors influencing SMBG checks and insulin administration. Overall, participants reported missing more glucose checks in the morning (59.4%) than in the afternoon (27.5%) or evening (13.2%). Participants also reported missing more insulin doses in the morning (74.1%) than in the afternoon (17.9%) or evening (8.0%) [271], suggesting that mornings may be particularly challenging for youth with T1D. This information, which may otherwise have been missed if collected in a laboratory setting, could potentially provide a unique target for more tailored interventions addressing challenges with morning routines.

##### Using Smartphone Apps and Phone-Based Systems to Improve T1D

The apps and phone-based programs often include educational components (eg, teaching T1D-related information, promoting problem-solving skills, reminders to participate in diabetes care behaviors, positive psychology interventions, and cognitive behavioral treatments) with the goal of improving T1D self-care and glycemic control [272-284]. These app and phone-based mHealth programs have been shown to be related to improved glycemic control (eg, lower HbA_1c_, mean blood glucose, mean fasting glucose, and postprandial glucose levels), increased frequency of SMBG testing, reduced frequency of hypoglycemia, improved quality of life, decreased disengagement coping, reduced parental intrusions in diabetes care for youth who checked glucose regularly, reduced urgent diabetes-related calls by school nurses, decreased hospitalizations, reduced emergency department visits, increased feelings of safety, increased confidence, decreased worry over hypoglycemia, and increased T1D self-care compliance [13,254,255,257,258,262,263,272,273,276-278,280,285,286]. For example, the SuperEgo system was designed to provide patients with individually tailored texts on topics such as stigma, burnout, stress, and sports and exercise with regard to T1D. They found that the intervention group maintained their HbA_1c_ level, whereas the control group had an increased HbA_1c_ level [272]. These tools can also be integrative such that participants receive momentary feedback about their T1D treatment based on the information they log in the app or phone system. For example, Bin-Abbas et al [276] found that youth in an intervention where patients sent glucose readings to their T1D management team and received feedback (eg, how to adjust insulin dosage to avoid dysglycemia) had improved T1D control.

Importantly, these studies have revealed that certain patient characteristics may make a child with T1D more likely to respond to phone-based systems. For example, Herbert et al [268] found that girls and patients who said they sent a large amount of personal texts in their daily lives were more likely to respond to a text message–based intervention. Furthermore, Bergner et al [275] found that youths with T1D found a text-based system for a positive psychology intervention more acceptable than phone call–based systems. However, although some studies have found that app- and phone-based program usage can improve T1D not all found statistically significant differences [282,287], and some found increased patient burden. For example, studies have shown increased conflict in families with app usage, including an increased perception of conflict about logging blood sugars, a sense of increased *nagging* from parents if the youths checked their glucose irregularly, decreased caring behaviors from parents, and increased unsupportive behaviors from parents [263,281].

Social app platforms have also been considered for better understanding and treating T1D in youths in their daily lives. For example, a study found that participants wanted to incorporate a social platform in a diabetes decision tool app [288], whereas another study used the Instagram social platform as a tool to collect data about what T1D looks like in daily life [289]. However, it is important to note that youth with T1D could also potentially feel pressured or discouraged by social media if they observe others posting about *perfect* blood glucose levels if they do not obtain similar readings. This is concerning as research has shown a relationship between comparison in social media and poor mental health [290]. Furthermore, there can be misleading information on social media about diabetes and health [291], which could potentially be harmful to self-care in youth.

#### Limitations of Smartphone Apps and Phone-Based Systems

The limitations associated with smartphone apps and phone-based systems are similar to those described for PDA. However, there are additional limitations. If a study uses a *bring-your-own-device* protocol, there may still be a sizable number of participants who do not own a device, resulting in selection bias if only individuals in higher economic groups have access to their own device. A *bring-your-own-device* platform might also require the development of different smartphone app versions (eg, iOS and Android) as participants may have a variety of phone types (eg, iPhone vs Android) that may not be compatible with the same app version. Finally, there may be concerns about damage to a participant’s own device or a study-provided device, such as cracked screens, that may interfere with data collection. On the other hand, if the study needs to provide a device for all participants, there could be study cost challenges.

#### Smartphone Apps and Phone-Based Systems Conclusions

Overall, smartphone apps and phone-based systems can provide a platform for collecting information that helps paint a more detailed picture of the daily experiences and challenges youth with T1D face that may not be otherwise captured reliably if assessed retrospectively in a laboratory or clinical setting. Thus far, numerous psychosocial and contextual factors have been found to be associated with T1D self-care behavior, such as fatigue, time of day, inaccessible devices, forgetfulness, and ignoring diabetes tasks. Taken together, this knowledge could potentially promote more defined interventional targets for improved glycemic control in youth with T1D (eg, developing different morning routines to promote self-care) that could potentially be delivered via an mHealth platform.

## Discussion

### Principal Findings

Overall, EMA has been used to better understand T1D in the daily lives of youth. EMA collection methods can provide significant advantages over in-lab testing, which may be confounded by phenomena such as recall bias and changes in behavior that result from the mere fact of being observed (eg, white-coat phenomenon) [12,202]. These methods also have the benefit of producing a much richer data set to better describe patterns of physiological and behavioral responses in T1D versus one-time snapshots.

EMA tools have already provided important information on care behaviors, physiological fluctuations, and complications of T1D that youth can experience in everyday life. CGMs have provided a great deal of information on glucose patterns, actigraphy has highlighted daily sleep challenges, and ABPM has shown the prominence of abnormalities in blood pressure and heart function in young people with T1D that can lead to other complications such as kidney disease. PDAs, smartphone apps, and phone-based systems have also uncovered numerous psychosocial and contextual environmental factors associated with T1D self-care (eg, wanting to fit into a group or time of day) and the negative consequences of glucose outside the recommended range (eg, cognitive impairment) [18,246,250]. Preliminary studies have also shown that smartphone apps and phone-based systems provide a potential platform for mHealth interventions for T1D and other conditions, such as impaired sleep [197,198].

Given the significant benefits of EMA, there is a great need for its expansion to study T1D-related factors in the daily lives of youth. Larger and more diverse study samples are of the utmost importance. Including individuals with newly diagnosed T1D could provide the opportunity for clinicians to intervene with behaviors that can lead to medical complications early before they are established versus trying to eliminate them later in the course of the disease. EMA methods may also provide an opportunity to reach populations currently underrepresented in research, such as those with lower socioeconomic status, racial or ethnic minority status, or those living in rural areas that may have less access to clinics and research facilities [292,293]. This is especially important because some of these populations have consistently been shown to have worse T1D-related outcomes [294]. Furthermore, EMA methods are less dependent on physical laboratory space, which may be an advantage in circumstances when there are barriers to access, such as during the COVID-19 pandemic or for patients who have barriers to travel to the laboratory.

Future research should also expand the use of combined EMA method systems. An area in which this combination could be particularly helpful is measuring the relationship between glucose patterns and T1D complications in daily life, such as cognitive impairment (eg, glucose measured via CGM or cognitive function measured via testing on a smartphone app). Given that acute T1D complications and chronic hyperglycemia have been associated with acute and long-lasting cognitive differences when measured in the laboratory [11] and the relationship between SMBG and cognitive impairment in Gonder-Fredrick’s EMA study [246], it is essential to better understand the relationship between daily glucose patterns measured via CGM and cognitive function in the real world, especially considering that youths spend about 25% of their time participating in school activities during the academic year [295,296], which requires substantial, ongoing cognitive effort using cognitive functions that can fluctuate throughout the day [297-301].

There may also be other fluctuating short-term complications associated with daily glycemic changes to be explored, such as vision. In-lab research has shown that short-term fluctuations in glucose can significantly alter nerve function and morphology in the eye [302]. Given that youths can have significant glucose variability [7], it is possible that they may experience daily vision changes. Research studies using EMA methods could be conducted to determine how frequently patients experience vision changes throughout the day and whether regular disruptions predict long-term complications such as retinopathy.

Furthermore, psychiatric conditions are more common in individuals with T1D than in those without T1D [303]. EMA methods can be used to gain better insight into the patterns of psychiatric symptoms in daily life. For example, one could collect repeated information about anxiety to determine whether its origin is T1D related, given that symptoms of glucose variability and feelings of anxiety can overlap (eg, shakiness and sweatiness because of hypoglycemia or because of nerves over a school exam). The timing of symptoms could also be evaluated to determine if there is a relationship between these symptoms and glucose fluctuations (eg, anxiety is higher with frequent swings in glucose). This information could potentially be used to aid therapists and medical care teams in tailoring behavioral interventions to help ameliorate symptoms when they are most severe.

More sophisticated analytical systems could also be used to better integrate data from different EMA modalities (eg, machine learning) for a more fully developed model of factors that predict T1D self-care behaviors or disease complications. The rapid development of new T1D care systems, such as the closed-loop system, makes this a unique time to conduct such combination EMA studies. EMA data collection could also be combined with mHealth interventional platforms to make treatment adjustments in real time, as youths appear to enjoy technology platforms. Furthermore, these technology-based interventions may provide motivation, self-efficacy, and adherence benefits [304]. The implementation of mHealth interventions while simultaneously collecting in-the-moment data could potentially help individualize treatment by providing a type of precision-based medicine.

### General Conclusions

In conclusion, as shown in the textboxes (Textboxes 1 and 2), EMA methods such as CGM, actigraphy, ABPM, PDAs, smartphone apps, and phone-based systems have unique strengths that can help the field better understand T1D in the daily lives of youth. Such an understanding could potentially lead to tailored interventions to improve quality of life and reduce the risk of short- and long-term complications of T1D. However, the field is still in its infancy and should be expanded in future research to address the limitations of each tool.

**Strengths of popular technological ecological momentary assessment tools to measure type 1 diabetes in the daily lives of youth, including continuous glucose monitoring, actigraphy, ambulatory blood pressure monitoring, personal digital assistants, and phone app, call, and text-based systems.**

Continuous glucose monitoring

- Increased amount of glucose data
- More comprehensive glucose patterns
- Improved diabetes management and outcomes
- Provides a sense of empowerment for youth

Actigraphy

- Inexpensive
- Noninvasive
- Objective; more valid than self-report
- More comprehensive view of sleep patterns
- Records numerous measures of sleep
- Captures sleep in the natural environment

Ambulatory blood pressure monitoring

- Provides large amounts of data
- Measures heart function across daily contexts
- Avoids confounding in-lab factors (eg, white-coat phenomenon)

Personal digital assistant

- Captures complex relationships between diabetes-related variables in real life
- Connects to the internet for data upload
- Organizes information

App, call, text

- Captures complex relationships between diabetes-related variables in real life
- Typically with a participant in real time
- Potential platform for treatment intervention

**Limitations of popular technological ecological momentary assessment tools to measure type 1 diabetes in the daily lives of youth, including continuous glucose monitoring, actigraphy, ambulatory blood pressure monitoring, personal digital assistants, and phone app, call, and text-based systems.**

Continuous glucose monitoring

- Expense and insurance difficulties
- Sensor discomfort
- Alarm fatigue
- Psychological toil (eg, anxiety)
- Measurement errors

Actigraphy

- Low specificity
- Imperfect measure of atypical sleep
- Not always comparable with polysomnography
- Variable device use across studies

Ambulatory blood pressure monitoring

- Potential obstacle in daily life
- Inaccurate readings

Personal digital assistant

- Validity concerns
- Testing compliance difficulties

App, call, and text

- Validity concerns
- Expensive equipment
- Hardware and software issues
- *Bring your own device* can lead to selection bias (eg, only recruiting those able to afford devices)
- *Bring your own device* can lead to data collection difficulties (eg, cracked screens)
- Testing compliance difficulties

## Abbreviations

ABPM: ambulatory blood pressure monitoring
CGM: continuous glucose monitoring
DKA: diabetic ketoacidosis
EMA: ecological momentary assessment
HbA_1c_: hemoglobin A_1c_
mHealth: mobile health
PDA: personal digital assistant
SMBG: self-monitored blood glucose
T1D: type 1 diabetes
WUSTL: Washington University in St. Louis
